# Novel Micro-LC-MS/MS Method for the Quantification of Tenofovir and Its Active Metabolite Tenofovir-Diphosphate in Biological Matrices for Therapeutic Drug Monitoring

**DOI:** 10.3390/ph18060899

**Published:** 2025-06-16

**Authors:** Isabela Tarcomnicu, Simona Iacob, Valentina Anuta, Emil Neaga, Dan Otelea

**Affiliations:** 1 National Institute for Infectious Diseases “Prof. Dr. Matei Bals”, 021105 Bucharest, Romania; simonaaiacob@yahoo.com (S.I.); emilneagabio@yahoo.com (E.N.); dotelea@mateibals.ro (D.O.); 2 Infectious Diseases Department, Faculty of Medicine, “Carol Davila” University of Medicine and Pharmacy, 020021 Bucharest, Romania; 3 Department of Physical and Colloidal Chemistry, Faculty of Pharmacy, “Carol Davila” University of Medicine and Pharmacy, 020021 Bucharest, Romania; valentina.anuta@umfcd.ro; 4 Innovative Therapeutic Structures Research and Development Centre (InnoTher), “Carol Davila” University of Medicine and Pharmacy, 6 Traian Vuia Street, 020956 Bucharest, Romania

**Keywords:** tenofovir, tenofovir diphosphate, active metabolite, liquid chromatography, mass-spectrometry, phosphatase inhibition, therapeutic drug monitoring

## Abstract

**Background/Objectives**: Sustained drug exposure is a key factor in the treatment of patients infected with human immunodeficiency virus (HIV) or hepatitis B virus (HBV) in order to achieve the intended virological response. Although influenced also by other parameters, adherence to the treatment scheme is the most important for adequate drug exposure. This can be assessed by therapeutic drug monitoring (TDM). Tenofovir (TFV) is a nucleotide analogue used in the treatment of both HIV and HBV. Although various analytical methods for the quantification of tenofovir prodrugs have been published, there is limited literature on methods for simultaneous TFV and its active metabolite, tenofovir diphosphate (TFVDP) direct determination. **Methods**: In this study, we describe a novel micro-liquid-chromatography-mass spectrometry (micro-LC-MS/MS) method for TDM of TFV and TFVDP in biological matrices (whole blood, plasma). The challenging separation of the high-polarity analytes was resolved on an amino stationary phase, eluted in HILIC (hydrophilic interaction liquid chromatography) mode. The sample preparation included a clean-up step with hexane for the removal of lipophilic compounds and then protein precipitation with organic solvent. **Results**: The achieved low limits of quantification in blood were 0.25 ng/mL for TFV, and 0.5 ng/mL for TFVDP. Linearity, accuracy (91.63–109.18%), precision (2.48–14.08), and stability were validated for whole blood matrix, meeting the guidelines performance criteria. Samples collected from treated patients were analyzed, with results being in accordance with the reported pharmacokinetics. **Conclusions**: The new method is adequate for analyzing samples in a clinical set-up. The measurement of both TFV and TFVDP improves clinical decision by an in-depth evaluation of long-term adherence, and together with viral load and resistance data helps guiding the treatment towards the intended virological suppression.

## 1. Introduction

Tenofovir (TFV) is an antiviral agent widely used in the treatment of patients infected with human immunodeficiency virus (HIV) or hepatitis B virus (HBV), and also for pre-exposure prophylaxis of HIV (PrEP). Structurally, TFV is a nucleotide analog that undergoes intracellular phosphorylation to form TFV-diphosphate (TFVDP), the pharmacologically active moiety [1,2]. TFV is available in two prodrug formulations: tenofovir disoproxil fumarate (TDF) and tenofovir alafenamide (TAF), that enhance the bioavailability of TFV, allowing for effective systemic absorption and subsequent conversion to its active form. Their pharmacokinetics in plasma was evaluated in different studies [3,4,5,6,7]. Compared with the parent drug, it was found that for nucleoside/nucleotide analogs the active metabolite possesses a prolonged intracellular half-life and may demonstrate distinct pharmacokinetic characteristics compared to its parent compound [1,8,9,10,11].

To accurately assess patient compliance, healthcare professionals employ a combination of non-pharmacological methods (including self-reports, pharmacy records, and pill counts) and pharmacological approaches (such as measuring plasma drug concentrations) [12]. Monitoring the plasma concentrations of antiretroviral (ARV) medications in patients undergoing treatment is crucial for therapeutic drug monitoring (TDM) and adherence assessment, which is essential for achieving a sustained virological response [13].

The distinctive pharmacokinetics of the active metabolites, with longer half-life, has recently been exploited in order to assess both short-term and prolonged exposure to ARVs (adequate parent drug but very low concentrations of metabolites may indicate drug administration only before the visit to the clinic) [2,12]. With a half-life of 17 days in red blood cells (RBC), TFVDP reflects better the long-term adherence [8,14,15]. Based on this approach, our group has also tested the analysis of phosphorylated metabolites for nucleoside/nucleotide analog ARVs as a tool to monitor long-term adherence.

Nowadays, LC-MS/MS has emerged as the gold standard for the quantitative analysis of drugs in biological matrices [16]. Several studies have been published with respect to TFV and TFV prodrugs measurement in plasma [3,4,5,6,7,17,18,19,20,21]. As for the active diphosphate metabolite, most of the reported methods focus on the analysis of isolated and lysed peripheral blood mononuclear cells (PBMCs) [1,22,23,24], with indirect quantification following the hydrolysis of the phosphate group, using calibration curves spiked with TFV. One article presents a method for direct TFVDP quantification in HBV-infected HepG2 hepatocytes [25]. In most of these studies, the analytical approach is based on reversed-phase separation, using high content of aqueous mobile phase in the initial conditions, in order to ensure enough retention for the very polar phosphates. More recent studies have explored the use of dried blood spots (DBS) as a suitable sample type for evaluation of patient adherence [14,26,27,28,29,30,31]. TFVDP was again measured indirectly, using the aforementioned approach [3]. The DBS samples are easy to collect, store and ship from the point of care to the centralized lab, therefore the use of this type of sample for HAART monitoring has expanded considerably [2,12,14,28,32,33,34,35,36]. The work with DBS has a few analytical drawbacks though: the volume of sample cannot be precisely measured (a punch is approximated at 3 µL blood) and the lint from the paper or other solid floating debris could clog some parts of the instrument (autosampler loop, tubing, electrode). Since we collect the samples within the hospital and therefore do not need to transport them, and because our laboratory is equipped only with a micro- liquid chromatography -MS/MS system, that is more prone to clogging from lint and other DBS-related impurities, we decided to use samples of whole blood instead of DBS. This manuscript presents, for the first time, the development of a sensitive and rapid hydrophilic interaction liquid chromatography (HILIC) method on a microbore column, for simultaneous quantification of TFV and TFVDP in whole blood and plasma. TFVDP is quantified directly in the biological sample, without hydrolysis to TFV, after inhibition of phosphatase activity at alkaline pH conditions.

## 2. Results and Discussion

### 2.1. Analytical Considerations

**Figure 1:**
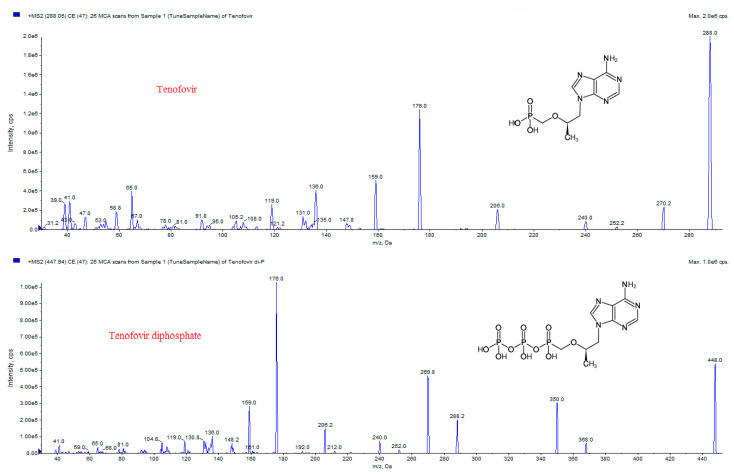
Chemical structures and MS/MS spectra of tenofovir and tenofovir diphosphate.

The high polarity of both TFV and TFVDP (chemical structures and MS/MS spectra in depicted in Figure 1), makes their chromatographic separation very challenging.

Our initial efforts were directed towards the determination of tenofovir, exploring a range of stationary phases and mobile phase combinations. Thus, Octadecyl (Halo C18, 50 × 0.5 mm, 2.7 µm, 90 Å), pentafluorophenyl propyl (Halo PFP, 50 × 0.5 mm, 2.7 µm, 90 Å), amide (Halo Amide, 50 × 0.5 mm, 2.7 µm, 90 Å), all from Advanced Materials Technology, were tested in reversed phase mode, maintaining a flow rate between 30–35 µL/min, with mobile phases based on water (A) and acetonitrile or acetonitrile/methanol (1/1, *v*/*v*) (B), containing organic modifiers like formic acid, ammonia or ammonium acetate.

However, these attempts consistently resulted in suboptimal outcomes, characterized by low retention, broad peaks, and poor peak shape.

Considering that hydrophilic interaction liquid chromatography (HILIC) is a viable alternative for polar compound separation, a Halo HILIC 50 × 0.5 mm, 2.7 µm, 90 Å was evaluated, but without improved results.

Subsequent experiments with an amide stationary phase in HILIC mode, using a mobile phase composed of water and acetonitrile containing 0.0125% ammonia led initially to good peak shape and retention. However, the retention was lost after the injection of plasma extracts.

The investigation ultimately identified the amino stationary phase (Luna NH_2_, 150 × 0.3 mm, 3.0 µm, 100 Å) as the effective choice in HILIC mode. The column allowed for adequate separation of TFV; further optimization of the gradient, upon inclusion of TFVDP, yielded the desired analytical results for both analytes, although the parameters were still critical (fresh mobile phase needed to be prepared each day).

**Figure 2:**
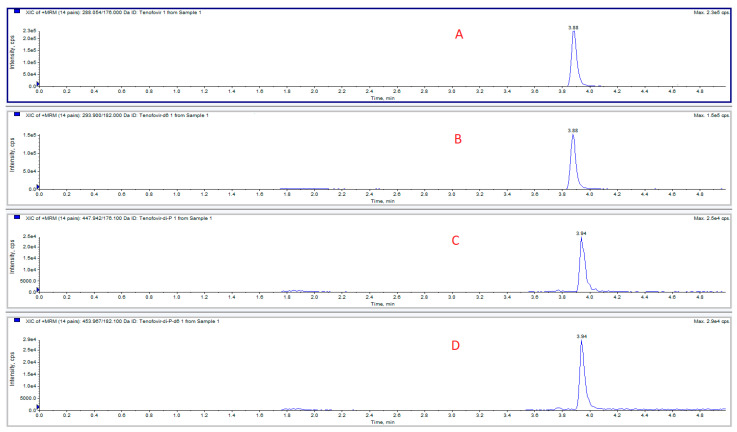
Chromatograms recorded on the MRM transitions of tenofovir (**A**) and tenofovir diphosphate (**C**) and their corresponding deuterated internal standards (d6) (**B**,**D**) in a 3 ng/mL solution. Column: Luna NH_2_, 150 × 0.3 mm, 3.0 µm, 100 Å; mobile phase—gradient of acetonitrile and water, with 0.025% ammonia. Injection volume: 2 µL.

The chromatogram of a standard mix is depicted in Figure 2.

### 2.2. TFVDP Stability

**Figure 3:**
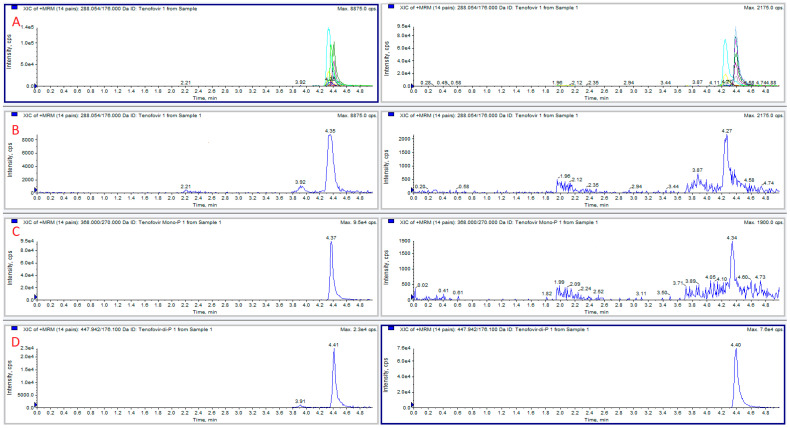
Extracted ion chromatograms overlay (**A**) recorded on the MRM transitions of TFV (**B**), TFVMP (**C**) and TFVDP (**D**) and their corresponding deuterated internal standards in an extracted sample (as described in paragraph 3.5). Blood sample diluted 1:5 with water and spiked with 520 ng/mL TFVDP in a non-stabilized sample (**left**) and with phosphatases inhibition (**right**). High peaks of TFMP were observed only after a short time (30 min) at room temperature without enzyme inhibition.

When preparing blood samples spiked with TFVDP, we have observed that the added diphosphate metabolite is unstable in blood, undergoing degradation to tenofovir monophosphate (TFVMP) and TFV. A series of experiments was conducted to evaluate this degradation by phosphatases and counteract it. TFVDP only (1 or 2 µg/mL) was added to blood or plasma; 50 µL aliquots were extracted in the presence of isotope-labeled internal standards, and the formation of TFV and TFVDP was monitored, together with TFVDP degradation. It was found that without stabilizers, significant amounts of TFVMP were produced from TFVDP in blood within 15 min, while in plasma the predominant degradation product was TFV. The extent of the phenomenon is illustrated in Figure 3. The first attempt to reduce phosphatases activity was performing all the sample preparation procedures on ice bath. No significant improvement was observed.

To mitigate the enzymatic degradation of TFVDP during sample handling, we systematically evaluated a panel of phosphatase inhibitors. These included disodium EDTA (10 mM), sodium orthovanadate (adjusted to pH 9), sodium and potassium tartrate, imidazole, and iodoacetic acid individually, and an in-house inhibitor cocktail containing orthovanadate, iodoacetic acid, tartrate, imidazole and sodium fluoride (80 mM each) at alkaline pH (further named “Inhibitor mix”). While disodium EDTA proved sufficient to stabilize TFVDP in plasma, its performance—along with that of the other inhibitors—was suboptimal in whole blood, where degradation remained significant. Notably, combinations of inhibitors did not yield additive or synergistic effects.

To address the pronounced instability observed in blood, we adopted a sample dilution strategy using ultrapure water (1:5, *v*/*v*), thereby reducing the cellular enzymatic load. This approach mirrored previous studies utilizing low-cell environments (e.g., 20 million cells/mL) for the quantification of nucleoside/nucleotide analogs [1,25]. Considering an average red blood cell (RBC) concentration of 5 million cells/µL, the resulting post-dilution value of ~1 million RBC/µL approximates those conditions, offering a practical compromise. Subsequent testing of the most promising inhibitors under these diluted conditions revealed the Inhibitor mix, as well as sodium orthovanadate alone, as a consistently effective agents in preventing TFVDP degradation. However, guided by published evidence that alkaline pH deactivates many phosphatases by altering the protonation state of catalytic residues and destabilizing enzyme conformation [37], we further hypothesized that increasing the sample pH might inactivate a broad range of phosphatases in our blood matrix. Therefore, blood samples were alkalinized using 1.25% ammonia (*v*/*v*), followed by freezing, thawing, and processing under standardized conditions. The results were comparable with those obtained with sodium orthovanadate (pH = 9), or with the Inhibitor mix. Without speculating that this method is better than other usual inhibitor cocktails, for our study alkaline pH adjustment was suitable and it was preferred because it is simple and avoids increasing the salt content of the samples.

**Figure 4:**
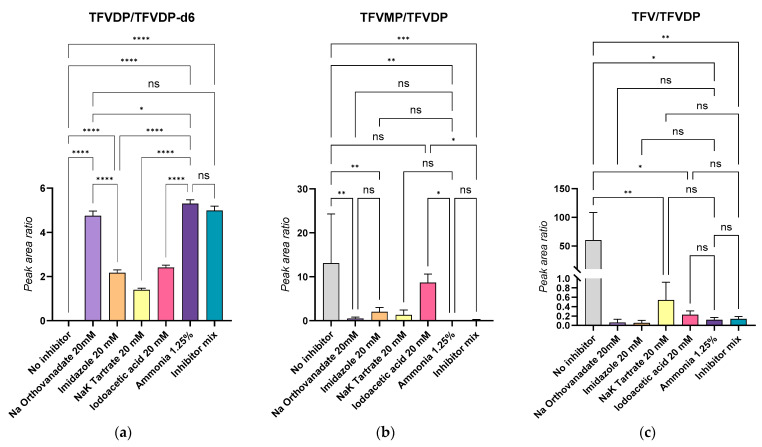
TFVDP stability in the presence of phosphatase inhibitors. Comparison of peak area ratios measured by LC-MS/MS after spiking blood samples with TFVDP (1 µg/mL), followed by enzyme inhibition, freezing, thawing, and processing after 30 min. The initial TFVDP/TFVDP-d6 ratio was 5:1. (**a**) Ratio of TFVDP to internal standard (TFVDP-d6); (**b**) TFVMP to TFVDP ratio; (**c**) TFV to TFVDP ratio. Error bars represent standard deviations (n = 4). Statistical significance: * *p* < 0.05, ** *p* < 0.01, *** *p* < 0.001, **** *p* < 0.0001, ns = not significant.

The outcomes of these experiments are summarized in Figure 4. In panel (a), the TFVDP/TFVDP-d6 peak area ratio was highest for ammonia-treated and orthovanadate-treated samples, indicating minimal degradation of TFVDP. Panels (b) and (c), which report the relative levels of the degradation products TFVMP and TFV, respectively, show that both ammonia and orthovanadate significantly suppressed metabolite breakdown (*p* < 0.0001), whereas other inhibitors such as imidazole, tartrate, and iodoacetic acid were markedly less effective.

Importantly, no significant difference was observed when using inhibitor combinations compared to ammonia or orthovanadate alone. Given its ease of use, compatibility with LC-MS/MS workflows, and low ionic strength, 1.25% ammonia was selected as the stabilizing agent for routine use in whole blood. Calibration curves and quality control samples were thus prepared in diluted blood stabilized with ammonia. For plasma, 10 mM disodium EDTA remained the inhibitor of choice due to its simplicity and effectiveness in a low-cell matrix.

### 2.3. Matrix Effects and Optimization of the Sample Preparation Protocol

Initially, methanol was selected for cell lysis and protein precipitation in both blood and plasma samples. Utilizing a classical precipitation approach with a high ratio of organic solvent to biological sample (7:1, *v*/*v*), a clean supernatant was obtained after a single centrifugation step. However, this method failed to recover TFVDP in the final extract probably due to its limited solubility in organic solvents or tendency to co-precipitate with the proteins, at high organic solvent concentrations. To address this, the precipitation process was modified to a two-step procedure, maintaining an organic solvent to water phase ratio of 7:3 (*v*/*v*). For the completion of precipitation and to obtain a clear extract, 200 µL of the initially unclear supernatant were transferred to clean 1.5 mL polypropylene tubes, with an additional 200 µL of methanol added. This adjusted protocol proved suitable for plasma samples, even following enzyme inhibition. Nevertheless, when the pH was adjusted to alkaline for blood samples, the resulting extracts the extracts were still very dirty. The precipitation solvent was switched to acetonitrile (instead of methanol), which provided adequate extracts.

Last but not least, an important matrix effect was noticed, with an up to 100-fold decrease in detector signal after injecting 4–5 biological extracts onto the column. Attempts to cleanse the column with an aqueous mobile phase were ineffective, directing us to the conclusion that ion suppression was caused not by retained polar compounds but by the accumulation of phospholipids in the column. This led to the necessity of a supplementary washing step during sample preparation. Attempts to remove lipids using hexane and chloroform on the methanolic extract were initially unsuccessful, as the lipids causing the matrix effect were soluble in both methanol and acetonitrile. Pre-precipitation washing of the biological sample with a non-polar solvent, particularly hexane, significantly diminished the ion suppression effect. Thus, after mixing with internal standards hexane was added first to the sample for lipid removal, after which the organic layer was discarded, and the process of protein precipitation was carried out as planned.

### 2.4. Concentration Range

The calibration ranges of the curves for quantifying TFV and TFVDP in blood was based on both existing literature references [1,12,14,22,23,24] and in-house initial findings from analyses of actual patient samples. Our approach was to express the analyte concentrations in ng/mL instead of ng per million cells. We used an estimate of 5 million RBC/µL and a volume of 3 µL blood/punch for comparing the obtained results with those reported in literature. To mitigate TFVDP degradation by phosphatases, diluted blood (1:5) was used as a matrix. Samples from patients were processed prior to LC-MS/MS using the same protocol as calibration curves (CCs) and quality controls (QCs); therefore, for the calculation of the final concentrations the measured values were multiplied by 5. This method, therefore, suffices for adherence assessment without the need for cell separation, even though different cell types were also isolated in this study, and prepared for analysis as per standard procedures (data not included in the manuscript).

For plasma, TFV concentration range was selected based on the existing literature data [1,3,6] and FDA drug label. No dilution was applied for plasma samples.

### 2.5. Method Validation

A complete validation was performed for the quantitative analysis of TFV and TFVDP from blood; a summary of results is presented in Table 1.

Adequate stability, analytical precision/accuracy and matrix effect were achieved with the optimized method. Samples were stable for 1 month at −30 °C, for 5 freeze–thaw cycles and for 24 h at 10 °C in the autosampler. Benchtop stability was evaluated for 4 h at room temperature with blood samples spiked with TFVDP only and the results were within 96.9 and 104.714 from nominal in stabilized conditions, while in the absence of the phosphatase inhibitor the degradation was, on average, 85% at all tested concentrations. Calibration curves were linear for both TFV and TFVDP, with correlation coefficients > 0.995.

### 2.6. Application of the Method for TDM

**Figure 5:**
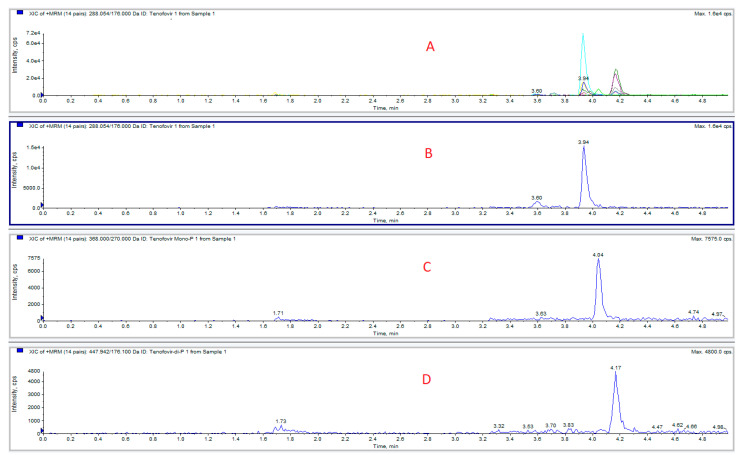
Extracted ion chromatograms overlay (**A**) recorded on the MRM transitions of TFV (**B**), TFVMP (**C**) and TFVDP (**D**) and their corresponding deuterated internal standards in an extracted sample from a treated patient (number 1). As it can be seen, all three peaks are present. TFV and TFVDP were quantified.

Blood samples were collected, with informed consent, from 11 HIV-infected patients treated with tenofovir, 12 to 24 h after dosing. Two patients (numbers 10 and 11) received tenofovir alafenamide 25 mg, the others being administered with tenofovir disoproxil fumarate 245 mg. A typical chromatogram obtained from a patient enrolled in the study is presented in Figure 5. The concentrations determined blood and plasma samples are shown in Table 2.

In blood, TFV ranged from below the limit of quantification (BLQ) to 60.135 ng/mL, and TFVDP from 15.23 to 396.41 ng/mL. Concentrations below the low limit of quantification were obtained for both analytes, in two patients. In plasma TFVDP was not detected (below the low limit of quantification—LLOQ) and TFV ranged from 13.526 to 232.004 ng/mL (Table 2).

For two of the patients (7 and 9), the blood sample was not available (n/a); only plasma was analyzed. However, therapeutic drug monitoring continues and we will use the newly developed method to asses TFVDP stability in patient samples and also to analyze and correlate the concentrations of TFV and its active metabolite in blood, plasma, red blood cells, PBMC and dry DBS, after developing specific matrix methods.

In patient 4, TFV concentration is higher than the metabolite concentration, thus suggesting an adherence issue (as mentioned in the introduction section, due to the longer half-life of TFVDP, adequate parent drug but very low concentrations of metabolites may indicate drug administration just before the visit to the clinic). Also, the low concentrations in both TFV and TFVDP for patient 10 suggest an adherence issue.

The levels measured in blood are in the range of those reported in the literature [2,12,14,28,35] and plasma concentrations corroborate with existing literature findings. Due to the absence of an analytical standard, TFVMP was monitored only qualitatively.

## 3. Materials and Methods

### 3.1. Chemicals

Reference standards of analytical grade—tenofovir (TFV), its active metabolite tenofovir-diphosphate (TFVDP, trimethylamine salt), and the isotopically labeled internal standards TFV-d6 and TFVDP-d6 (trimethylamine salt)—were sourced from Toronto Research Chemicals (Toronto, ON, Canada). HPLC-grade methanol and acetonitrile, along with analytical-grade 25% aqueous ammonia and hexane, were obtained from Merck (Darmstadt, Germany). Dimethyl sulfoxide (HPLC grade) was supplied by Scharlab (Sentmenat, Spain), whereas analytical-grade ammonium acetate and formic acid were procured from Lach-ner (Neratovice, Czech Republic).

Disodium EDTA was purchased from Serva Electrophoresis (Heidelberg, Germany). Sodium orthovanadate, sodium molybdate, sodium fluoride, sodium and potassium tartrate, imidazole and iodoacetic acid were purchased from Sigma-Aldrich (St. Louis, MO, USA).

Ultrapure water was produced with a Milli-Q purification system (Millipore, Molsheim, France).

### 3.2. Standard Solutions

TFV standard solution was prepared at 1 mg/mL in water/methanol (1:1, *v*/*v*) with 0.05% NaOH 1 M, while TFV-d6 was dissolved at 1 mg/mL in water. Following suitable salt correction, TFVDP and TFVDP-d6 were prepared also in water at 3 and 1 mg/mL, respectively. All solutions were stored at −30 °C. Working dilutions in methanol were obtained from the stock solutions when needed.

### 3.3. Calibration Curves and Quality Control Samples Preparation

Spiked calibration standards (CC) and quality control (QC) samples were prepared in whole blood diluted 1:5 with ultrapure water containing 1.25% ammonia (*v*/*v*). TFV concentrations were as follows: 0–0.25–1–4–16–64–128–256 ng/mL (CC) and 0.75–15–210 ng/mL (QC Low, Medium and High, respectively). TFVDP concentrations were as follows: 0–0.5–2–8–32–128–256–512 ng/mL (CC) and 1.5–30–420 ng/mL (QC Low, Medium and High, respectively).

For the quantification of the analytes in plasma (containing 10 mM EDTA), calibration curves were built with TFV at 0–0.4–2– 10–50–250–500 ng/mL (CC) and 1.2–36–360 ng/mL (QC Low, Medium and High, respectively). Spiked TFVDP concentrations in plasma were 0–0.8–4–20–100–500–1000 ng/mL (CC) and 2.4–72–720 ng/mL (QC Low, Medium and High, respectively).

### 3.4. Blood Samples for Adherence Evaluation

Blood samples were collected from HIV-positive patients at the National Institute for Infectious Diseases “Prof Dr. Matei Bals”, with the approval of the Bioethical Committee (protocol code C10242/02.10.2024, approved 18 October 2024). All patients were under treatment with anti-retroviral drugs, and sampling was performed from 12 to 24 h after dosing.

Blood samples (4 mL each) were collected using potassium EDTA (sampling tubes EDTAK_3_, 4.0 mL, Englober SRL, Brasov, Romania) or sodium citrate (9NC sampling tubes, BD, Plymouth, UK) as anticoagulants. Aliquots of 0.5 mL were used for plasma separation, by centrifugation (5 min, 3900 rpm), within 60 min from sampling. Other aliquots (0.4 mL) were transferred directly into cryo-tubes and diluted 1:5 with ultrapure water containing 1.25% ammonia (*v*/*v*). The rest of the sample was used for PBMC separation (PBMC data not presented here). All samples were frozen at −30 °C until analyzed.

### 3.5. Sample Preparation Procedures Prior to LC-M/MS

#### 3.5.1. Whole Blood Sample Clean-Up

Aliquots of 50 µL of blood (CCs, QCs, and patient samples) were dispensed into 1.5 mL Eppendorf tubes, and 50 µL internal standard mixture (TFV-d6 100 ng/mL and TFVDP-d6 200 ng/mL in H_2_O) was added in each tube. Following a gentle mix, 300 µL of n-hexane was introduced, and the tubes underwent vortex mixing for 5 min at medium speed, followed by centrifugation for 3 min at 14,000× *g*. The organic layer was subsequently discarded.

#### 3.5.2. Protein Precipitation

Following the hexane clean-up step of the whole blood samples, 200 µL acetonitrile/water (70:30, *v*/*v*) were added in each tube, followed by vortex-mixing (5 min at medium speed) and centrifugation (5 min at 15,000× *g*). Aliquots of 200 µL of the supernatant were transferred into clean 1.5 mL Eppendorf tubes, to which another 200 µL of acetonitrile was added, followed by vortex mixing and centrifugation as aforementioned. Finally, 180 µL of the clear supernatant was pipetted in 96-well polypropylene microtiter plates (Greiner Bio-One, Kremsmünster, Austria) for LC-MS/MS analysis.

Blank blood was frozen at −80 °C before use; therefore, cells were lysed. CCs and QCs were built in blood diluted 1 + 4 with water and alkalinized with 1.25% ammonia, for phosphatases inhibition. Patient samples received identical treatment before undergoing the sample preparation protocol, ensuring uniform composition across samples.

Plasma was processed according to the same protocol, but protein precipitation was achieved with methanol/water (70:30, *v*/*v*) in the first step and pure methanol in the second. In the final step 90 µL of clear supernatant were transferred in the microtiter plate and further diluted with 90 µL acetonitrile.

The equipment employed in the sample preparation procedures included an Eppendorf centrifuge 5180R (Eppendorf, Hamburg, Germany), a Corning LSE vortex mixer (Corning Inc., Corning, NY, USA), an analytical balance Kern ABT 100-5M, and a technical balance Kern EG (Kern&Sohn, Balingen, Germany).

### 3.6. Liquid Chromatography-Mass Spectrometry

TFV and TFVDP extracted from biological samples were separated on a microbore amino column (Luna NH_2_, 150 × 0.3 mm, 3.0 µm, 100 Å, Phenomenex, Torrance, CA, USA), maintained at 35 °C. The LC-MS/MS system comprised of an Ekspert™ micro-LC 200 system from Eksigent Technologies (Dublin, CA, USA), equipped with HTS PAL autosampler (CTC Analytics, Zwingen, Switzerland), and interfaced with a SCIEX Qtrap 5500 triple quadrupole-linear ion trap mass spectrometer (Sciex, Toronto, ON, Canada) equipped with a 50 µm electrospray microprobe. The column was eluted in HILIC mode, at a flow rate of 10 µL/min, in gradient, with a mobile phase consisting of water with 0.025% ammonia (A), and acetonitrile with 0.025% ammonia (B). The injection volume was 2 µL. The samples were kept in the autosampler stack cooled at 10 °C.

Analyses were performed in positive electrospray ionization mode. The chromatographic trace 288.054/176.0 was used for TFV quantification, while TFVDP was monitored on the trace 447.942/176.0. Mass transitions 288.054/135.9 and 447.942/270.0 were also used for confirmation of the measured compounds. Mass spectrometric parameters for the intermediate tenofovir monophosphate (TFVMP) were also optimized using extracted biological sample, and its characteristic transitions were acquired in order to study the degradation of TFVDP in blood and plasma. The mass spectrometric parameters are presented in Table 3.

The eluent from the HPLC column was introduced in the MS interface without splitting; research grade nitrogen served as both curtain and CAD collision gas, whereas zero-grade air maintained at 45 psi supplied the auxiliary and nebulizer gas flows. The electrospray source temperature was kept at 450 °C.

The optimization of the mass-dependent parameters for the analytes and their internal standards, both in positive and negative ion modes, was carried out by infusing directly in the TurboIonspray interface the compounds, diluted at 1 μg/mL (or less if needed) in water/methanol (1:1, *v*/*v*), with the help the syringe pump incorporated into the mass spectrometer. Interestingly, although the molecules contain electronegative phosphate groups, the best sensitivity, needed to measure the very low concentrations present in the biological samples, was achieved in positive mode.

### 3.7. Validation Procedures

The method for quantifying TFV and TFVDP in whole blood was validated in terms of selectivity, linearity, accuracy, precision, limits of quantification, sample stability under storage and processing conditions, extraction recovery, and matrix effects, in accordance with the April 2022 ICH guidelines, subsequently adopted by the FDA in November 2022 [38]. The analytical ranges to be validated were chosen based upon the expected concentrations as found in literature [1,2,4,19,20,21,22,23,24,26,28,34] and from in-house preliminary experiments. Matrix effects were evaluated comparing the signal of theoretical QC concentrations prepared in mobile phase (taken as 100% recovery) against those obtained after spiking blank extracts at identical concentrations. Matrix factors were calculated for each analyte, and isotope-labelled internal standards were employed to correct for residual matrix influence [39].

## 4. Conclusions

This manuscript describes the development and validation of a micro-LC-MS/MS method for the simultaneous determination of TFV and its active metabolite, TFVDP, in whole blood. To our knowledge, for the first time, the concentrations of TFVDP were determined directly on calibration curves spiked in matrix, phosphatases activity being effectively inhibited.

The chromatography in HILIC mode offered suitable retention and sensitivity for the highly polar compounds. As a drawback, we noticed pressure build-up to a somewhat higher extent than in the case of reversed-phase chromatography; nevertheless, more than 1500 biological extracts were able to be analyzed on a micro-column. The micro-LC set-up was the only one available at this time in our laboratory; however, the separation can be adapted for any other conventional or ultra-high-pressure system. Biological sample preparation is also easy and does not require expensive consumables and reagents. The micro-LC set-up consumes little mobile phase volume, thus being cheaper and more environmentally friendly than conventional set-ups. The achieved LLOQ of 0.4 ng/mL in plasma is comparable with that of the most sensitive published methods [4,7].

As for the whole blood, there is no method published so far, to our knowledge, and the LOQ of 0.25 mg/mL TFV/0.5 ng/mL TFVDP achieved with this method was enough to get comparable results with the methods using DBS.

The method was applied in the clinical laboratory for monitoring the adherence of HIV-infected patients. Concentrations obtained in real samples were in accordance with the published literature. This approach gives more accurate results, using precisely measured liquid samples, but, on the other hand, this represents a limitation, because the samples need to be sent to the laboratory immediately, or diluted with ultrapure water containing 1.25% ammonia according to the protocol and frozen before storage and transportation, thus it is not very easy to apply to remote point-of-care set-ups, like the DBS approach is.

Future studies will be carried out on blood, plasma, PMBC and DBS, to make correlations between concentrations of the antiviral drug and its metabolite in different types of biological samples and between drug concentrations and virologic response. The correlations between the TFV and TFVDP concentrations, together with viral load and resistance data, will be used to improve the clinical decision.

This approach is fast and straightforward for blood and plasma and it can be extended and validated also for other types of samples, like PBMC or dried blood spots.

## Figures and Tables

**Table 1:** Validation data for the LC-MS/MS method of simultaneous quantification of TFV and TFVDP.

Stability/Storage Conditions	Sample	TFV (ng/mL)	TFVDP (ng/mL)
Blood at −30 °C for 1 month	QC Low (n = 4)	103.50	92.45
	QC High (n = 4)	112.26	105.81
Blood freeze-thaw 5 cycles	QC Low (n = 4)	113.53	110.63
	QC High (n = 4)	111.27	95.37
Blood 4 h room temp.	QC Low (n = 4)	101.59	104.71
	QC High (n = 4)	100.65	96.90
Sample extracts 24 h 10 °C	QC Low (n = 4)	94.4	105.45
	QC High (n = 4)	90.7	94.29
**Precision and Accuracy**			
Within-run mean accuracy (%)	QC LOQ (n = 4)	117.31	93.03
	QC Low (n = 4)	102.45	99.84
	QC Medium (n = 4)	109.18	98.49
	QC High (n = 4)	100.65	91.63
Within-run precision (RSD %)	QC LOQ (n = 4)	14.08	10.86
	QC Low (n = 4)	4.35	13.78
	QC Medium (n = 4)	4.38	2.48
	QC High (n = 4)	5.18	5.84
Between-run mean accuracy (%)	QC LOQ (n = 12)	115.04	95.20
	QC Low (n = 17)	103.98	100.91
	QC Medium (n = 18)	108.08	92.9
	QC High (n = 18)	103.89	90.30
Between-run precision (RSD %)	QC LOQ (n = 12)	9.07	8.77
	QC Low (n = 18)	5.51	7.59
	QC Medium (n = 18)	4.38	5.28
	QC High (n = 18)	5.85	4.01
Extraction recovery (%)	QC Low (n = 4)	39.87	35.58
	QC Medium (n = 4)	42.88	38.78
	QC High (n = 4)	46.2	45.31
Matrix effect	QC Low (n = 4)	0.52	0.70
	QC Medium (n = 4)	0.55	0.79
	QC High (n = 4)	0.59	0.79

**Table 2:** TFV and TFVDP levels in clinical samples.

Patient Code	TFV (ng/mL)		TFVDP (ng/mL)
	Blood	Plasma	Blood
1	60.14	66.19	171.84
2	29.78	47.47	135.22
3	37.36	54.57	163.65
4	44.12	53.22	15.23
5	60.05	70.61	91.28
6	135.00	37.33	317.50
7	n/a	232.00	n/a
8	44.42	62.74	396.41
9	n/a	15.14	n/a
10	BLQ	13.53	18.95
11	2.76	28.92	38.03

**Table 3:** Mass spectrometric parameters for quantitation of TFV and TFVDP.

Compound ID	Q1 Mass	Q3 Mass	CE	CXP
Tenofovir 1	288.054	176.0	37	12
Tenofovir 2	288.054	135.9	35	16
Tenofovir-d6 1	293.9	182.0	37	12
Tenofovir-d6 2	293.9	164.0	40	12
Tenofovir di-P 1	447.942	176.1	59	10
Tenofovir di-P 2	447.942	270.0	37	16
Tenofovir di-P-d6 1	453.967	182.1	61	10
Tenofovir di-P-d6 2	453.967	294.1	37	14
Tenofovir mono-P 1	368.0	270.0	36	16
Tenofovir mono-P 2	368.0	288.0	35	16
Tenofovir mono-P-d6 1	374.0	276.0	35	16
Tenofovir mono-P-d6 2	374.0	294.0	35	16

## Data Availability

The original contributions presented in the study are included in the article, further inquiries can be directed to the corresponding author.
